# Biological Functions and Analytical Strategies of Sialic Acids in Tumor

**DOI:** 10.3390/cells9020273

**Published:** 2020-01-22

**Authors:** Xiaoman Zhou, Ganglong Yang, Feng Guan

**Affiliations:** 1 The Key Laboratory of Carbohydrate Chemistry and Biotechnology, Ministry of Education, School of Biotechnology, Jiangnan University, Wuxi 214122, China; 2 Joint International Research Laboratory of Glycobiology and Medicinal Chemistry, College of Life Science, Northwest University, Xi’an 710069, China

**Keywords:** sialic acid, sialyltransferase, sialidase, tumor sialylation, sialic acid labeling

## Abstract

Sialic acids, a subset of nine carbon acidic sugars, often exist as the terminal sugars of glycans on either glycoproteins or glycolipids on the cell surface. Sialic acids play important roles in many physiological and pathological processes via carbohydrate-protein interactions, including cell–cell communication, bacterial and viral infections. In particular, hypersialylation in tumors, as well as their roles in tumor growth and metastasis, have been widely described. Recent studies have indicated that the aberrant sialylation is a vital way for tumor cells to escape immune surveillance and keep malignance. In this article, we outline the present state of knowledge on the metabolic pathway of human sialic acids, the function of hypersialylation in tumors, as well as the recent labeling and analytical techniques for sialic acids. It is expected to offer a brief introduction of sialic acid metabolism and provide advanced analytical strategies in sialic acid studies.

## 1. Introduction

The term “sialic acid” first appeared in 1952 to describe *N*-acetylneuraminic acid, a major product released by mild acid hydrolysis of glycolipids in the brain or salivary mucins [1,2]. Sialic acids are a subset of nine carbon acidic sugars that contain approximately fifty derivatives of neuraminic acids. The most common sialic acid derivatives found in mammals are *N*-acetylneuraminic acid (Neu5Ac) and *N*-glycolylneuraminic acid (Neu5Gc). Neu5Ac has an acetyl group on the fifth carbon atom (C5) while Neu5Gc has a glycolyl group instead. Interestingly, humans lack Neu5Gc caused by the mutation of the cytidine monophosphate *N*-acetylneuraminic acid hydroxylase (*CMAH*) gene that codes the enzyme transforming CMP-Neu5Ac to CMP-Neu5Gc [3,4,5]. However, Neu5Gc is still found in the human glycome as it can be obtained through dietary sources [6]. In this review, “sialic acid” refers to Neu5Ac unless specifically emphasized.

**Figure 1:**
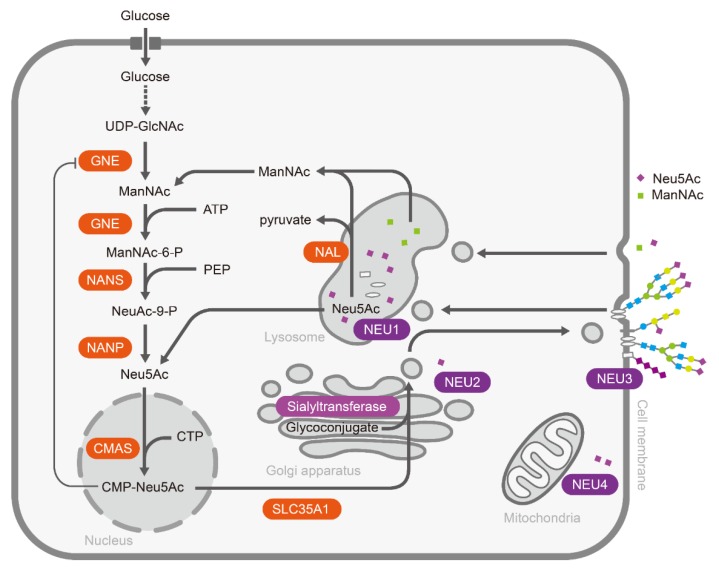
The metabolic pathway of sialic acids in mammalian cells.

In general, sialic acids normally exist as the terminal sugars in the periphery of oligosaccharides via different glycosidic linkages (α2,3, α2,6 α2,8, and α2,9) [7]. Polysialic acid (PSA), a linear homopolymer of sialic acids mainly in α-2,8 and/or α-2,9 linkages, is usually attached to glycoproteins, such as the neural cell adhesion molecule (NCAM) [8,9]. Sialic acid metabolic pathways include the cooperation of certain enzymes that catalyze the biosynthesis, activation, and transfer of sialic acids to glycoconjugates, as well as the removal and degradation of sialic acids (Figure 1) [10]. In the human body, sialic acid biosynthesis starts at UDP-GlcNAc in the cytosol. Primitively, sialic acid synthesis is catalyzed by UDP-GlcNAc 2-epimerase/ManNAc-6-kinase (GNE), converting UDP-GlcNAc to ManNAc-6-P [11,12]. Next, ManNAc-6-P is transformed to Neu5Ac by Neu5Ac 9-phosphate synthase (NANS) with phosphoenolpyruvate (PEP) and Neu5Ac-9-phosphate phosphatase (NANP) [13,14]. Finally, Neu5Ac synthesized in cytosol is transferred to the nucleus and activated by the cytosine 5′-monophosphate *N*-acetylneuraminic acid synthetase (CMAS) to form CMP-Neu5Ac [15]. 

After activation, CMP-Neu5Ac is transferred to the glycoconjugates in the Golgi apparatus by a family of linkage-specific sialyltransferases. About 20 sialyltransferases have been cloned and characterized, including ST3GAL1-5, ST6GAL1, 2 and ST6GALNAC1-6, and ST8SIA1-6, which link Neu5Ac via its second carbon (C2) to the C3, C6 positions of other carbohydrates or the C8, C9 positions of another sialic acids, generating α2,3-, α2,6-, α2,8, or α2,9-linked sialic acids, respectively [7]. Finally, sialylated glycoconjugates are transported to the cell membrane or packaged for secretion. On the other side, sialic acids on glycoconjugates can be released by sialidases (also termed as neuraminidases). There are four mammalian sialidases, NEU1–4. Lysosomal sialidase NEU1 initiates the degradation of sialoglycoconjugates [16,17,18]; cytosolic sialidase NEU2 exhibits highest activity with gangliosides [19,20]; the plasma membrane-associated sialidase NEU3 is specific for gangliosides [21]; and sialidase NEU4, which is bound to the outer mitochondrial membranes via protein-protein interactions or occurs in the lysosomal lumen, has a wide substrate specificity from glycoproteins to gangliosides and oligosaccharides [22]. The released sialic acids are pumped back into the cytosol, where they can enter another cycle of sialyl glycoconjugate production or be broken down by *N*-acetylneuraminate lyase (NAL) to ManNAc and pyruvate [23]. Interestingly, Neu5Ac or ManNAc enter cells mainly through the pinocytic/endocytic pathways due to the lack of a specific transporter on the cell membrane [24].

In the following, we summarize the functions of sialic acids during tumor progression, especially in immune escape, tumor proliferation and metastasis, tumor angiogenesis and apoptosis resistance. Moreover, we also introduce the prevalent analytical methods, including the sialic acid quantification and labeling strategies by bio-affinity, the chemical reaction, and metabolic labeling.

## 2. Functions of Sialic Acids in Tumor Biology

**Figure 2:**
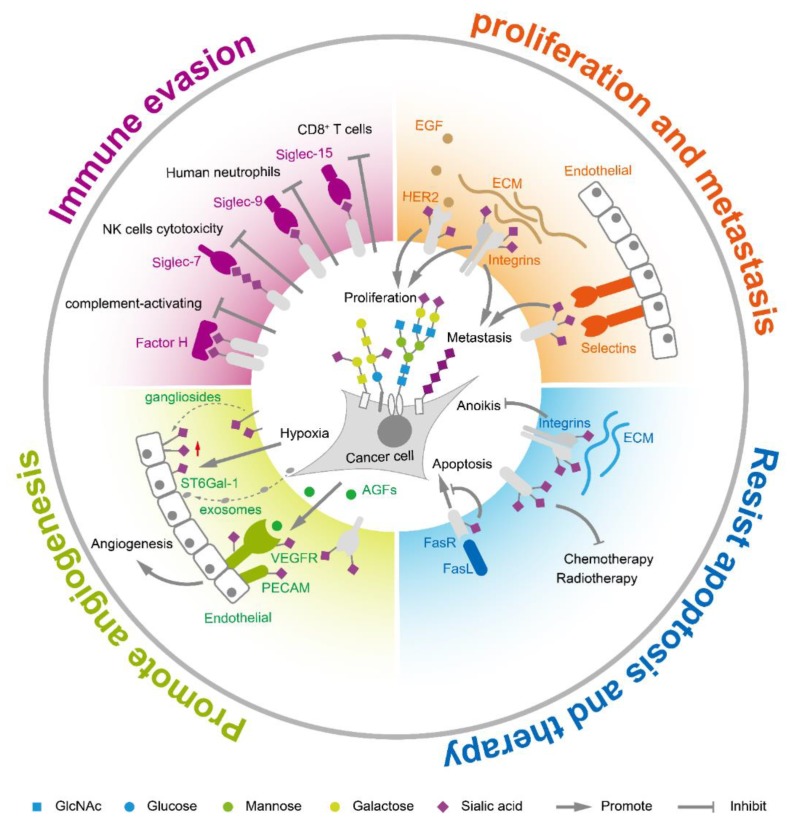
The functions of sialic acids in tumor biology.

Sialic acids on tumor cell surfaces are aberrantly expressed during tumor transformation and malignant progression. In general, hypersialylation is frequently observed in tumor tissues compared to corresponding normal tissue [25]. The total sialic acid in serum or glycolipid-bound sialic acids is significantly elevated in multiple cancers such as ovarian cancer, leukemia, colorectal cancer, and breast cancer [26]. Polysialic acid is found at high expression levels on several types of cancer including glioma [27,28,29], neuroblastoma [30], and lung cancer [31]. Hypersialylation helps to accelerate cancer progression and leads to poor prognosis. The increased sialic acids in tumor cells are mainly caused by the special metabolic flux and aberrant expression of sialyltransferases/sialidases. Generally, tumor cells increase the uptake of glucose, the raw material for sialic acid synthesis. It was reported that sialic acid metabolism was upregulated in highly metastatic breast tumors, while knocking out *CMAS* gene, a key node in sialic acid metabolism, inhibited the synthesis of the activated form of sialic acid and decreased the formation of lung metastases in vivo [32]. On the other hand, the aberrant expression of sialyltransferases and sialidases accelerated and sustained sialylation status on glycoconjugates. The sialylation further facilitates immune escape, enhances tumor proliferation and metastasis, helps tumor angiogenesis, and assists in resisting apoptosis and cancer therapy (Figure 2).

### 2.1. Sialic Acids Facilitate Immune Escape

Growing evidence suggests that sialic acids control the immune homeostasis and weaken immune activation in order to avoid or limit the damage of sialylated cells [33]. Sialic acids act as self-associated patterns to maintain the baseline of innate immune cells [34]. Sialic acid recognizing receptors are the main molecules to transmit the inhibitory signals to the immune system. Highly coated sialyl glycans on tumor cells interacted with these sialic acid receptors to escape immune surveillance. There are three groups of sialic acid receptors: selectins, factor H, and the family of sialic acid-binding immunoglobulin-like lectins (siglecs). The selectin family, including E-selectin, L-selectin and P-selectin, is related to tumor-associated inflammation [35]. Factor H is a central regulatory protein in the alternative complement pathway and binds α2,3-linked sialyl glycans in its C-terminal domain [36]. The complement pathway is a branch of the innate immune response that consists of numerous proteins that rapidly respond to microbial intruders, initiating the release of inflammatory mediators, phagocytic responses, and cell lysis. Sialyl glycans were suggested to prevent the activation of the complement system by recruitment of the complement control protein factor H to cell surface [37,38,39]. Siglecs are type I transmembrane proteins containing a sialic acid-binding site at N-terminus, and most of them own one or more immunoreceptor tyrosine based inhibitory motifs at C-terminus. The binding of sialic acid ligands to immune inhibitory siglecs results in immune evasion [40,41,42]. For example, blocking siglec-2 (CD22), an inhibitory B cell receptor specifically recognizing α2,6 sialic acids, increased tumor sensitivity towards immunotherapy [43,44,45]. Similarly, inhibiting siglec-7 and siglec-9 could protect tumor cells from NK cell responses [46]. Sialyl ligands for siglec-9 on tumor cells inhibited neutrophil activation [47]. CD24 on tumor cells interacts with siglec-10 on tumor-associated macrophages to promote immune evasion [48]. Blocking siglec-15 or siglec-9 disinhibited T cell activities and reduced tumor growth [49,50,51]. Focusing the immune inhibitory function of sialic acids and targeting sialic acid receptors offer potential important immunotherapy in cancer [52].

### 2.2. Sialic Acids Enhance Tumor Proliferation and Metastasis

Expression of sialylglycans are positively correlated with aggressiveness and metastasis in many cancers. Altered expression of sialylglycans is associated with epithelial-mesenchymal transition (EMT), an essential step for tumor progression and metastasis [53]. Transforming growth factor-β (TGF-β) induced EMT process caused upregulation of various sialyltransferases such as ST3GAL1, ST3GAL2, ST6GAL1, ST6GAL2, ST8SIA1, ST8SIA2, and ST8SIA4 [54,55,56,57,58,59,60,61]. The upregulation of those sialyltransferases resulted in accumulation of sialylglycans on the cell surface, assisting tumor cells in surviving and metastasis. α2,6-sialylation in hepatocellular carcinoma activated Wnt/β-catenin signaling to promote tumor cell proliferation, migration, and invasion [62]. Increased α2,6-sialylation on the human epidermal growth factor receptor 2 (HER2) facilitated gastric cancer progression via the Akt and ERK pathways [63]. Sialylation on the endothelial growth factors receptor (EGFR) was regulated by ST6GAL1 via the PI3K/Akt pathway [64], and inhibition of ST6GAL1 induced EGFR desialylation and anti-proliferation [65]. The α2,6-sialylated integrin α5β1 modulated FAK signaling and cell adhesion [66]. Polysialic acid controls tumor cell growth and differentiation by interfering with NCAM signaling at cell–cell contacts, as well as facilitates tumor invasion and metastasis [27,58,60,67,68]. Inhibiting polysialyltransferases ST8SIA2 and ST8SIA4 decreased polysialylation of NCAM, resulting in delayed metastasis in a xenograft rhabdomyosarcoma tumor mouse model [69]. Moreover, the sialyl glycans coated on tumor surface also contributed to their colonization during metastasis. For example, the enhanced sialylation, acting as ligands of selectin, which are vascular adhesion molecules, was associated with cancer progression and helped the adhesion and extravasation during metastasis [70,71,72,73,74].

### 2.3. Sialic Acids Promote Tumor Angiogenesis

Angiogenesis is the formation of new blood vessels from pre-existing ones, which is accurately controlled during embryonic development and wound repair. Around the microenvironment where tumor cells grow rapidly, new blood vessels are needed to meet the oxygen and nutrient requirements. Angiogenesis is stimulated by the angiogenic growth factors (AGFs), which are released by inflammatory cells or tumor cells. Among them, the most important AGF is the vascular endothelial growth factor (VEGF) family that includes VEGF-A, B, C, D, E, and placental growth factor [75]. VEGF affects angiogenesis by the interaction with polysialic acid [76]. Tumor cells grow in a hypoxia environment where sialic acids play an important role in tumor angiogenesis. Sialylation status could affect the growth factor–receptor interactions and related signal transduction in angiogenesis [77]. Gangliosides, one type of sialyl-glycosphingolipid, can be incorporated into the membrane of endothelial cells, increasing cell responsiveness to AGFs [78,79]. On the other hand, tumor cells can also regulate the sialic acid expression of surrounding cells by delivering ST6GAL1 through exosomes [80]. *N*-glycans with terminal α2,6-sialylation on the receptors of blood vessels such as VEGFR2 are required for the VEGF engagement and proangiogenic activation of endothelial cells [81,82]. α2,6-sialylation mediates the homophilic interaction of the platelet endothelial cell adhesion molecule (PECAM). Sialylated PECAM interacted with two other sialylated receptors, VEGFR2 and integrin β3, while the inhibition of sialylation in *ST6GAL1*^−/−^ mice prevented PECAM-VEGFR2 interaction on the endothelial surface, inducing endothelial cell apoptosis and inhibiting angiogenesis [83].

### 2.4. Sialic Acids Assist to Resist Apoptosis and Cancer Therapy

Sialic acids affect cell apoptosis mainly through two pathways, the Fas receptor-Fas ligand (FasR-FasL) apoptotic pathway and anoikis. FasR-FasL interaction, induced through activated T cells and mediated by caspase activation, is important for homeostasis of cells in the immune system and for immune-privileged site maintenance in the human body [84]. Sialylated FasR blocked the binding of Fas-associated adaptor molecules to the FasR death domain, thus inhibiting the formation of the death-inducing signaling complex [85]. Sialyltransferase ST6GAL1 was found to elevate α2,6-sialylation on FasR to inhibit the apoptotic signaling in colon carcinoma cells [85]. Moreover, α2,6-sialylation impaired internalization of the Fas receptor and prevented further positive feedback loops for Fas-mediated apoptosis [86]. Anoikis is another cell death pathway induced by cell detachment from extracellular matrix (ECM). It is a vital mechanism in preventing adherent-independent cell growth and attachment to an inappropriate matrix [87]. Cancer cells showed a higher degree of anoikis resistance than the normal intestinal epithelial cells [88,89]. Integrin-mediated cell-ECM interactions are functionally involved in regulating tumor angiogenic response during cancer metastasis. The loss of integrin-mediated epithelial cell-ECM interactions decreases the phosphorylation of down streaming effectors such as FAK, PI3-K, ERK1, and MAP kinases, thus mediating cell susceptibility to anoikis [90]. However, α2,6-hypersialylation of fibronectin receptor integrin α5β1 could avoid anoikis by preventing galectin-1 binding to integrins [91,92]. On the other hand, α2,6-sialylation showed vital effects on therapeutic resistance in many cancers [93,94,95], possibly through the sialylated receptors such as EGFR [96,97,98]. EGFR sialylation was reported to suppress its dimerization and to induce phosphorylation, reducing the effects of tyrosine kinase inhibitors [98].

## 3. Sialic Acid Analysis Technology

**Figure 3:**
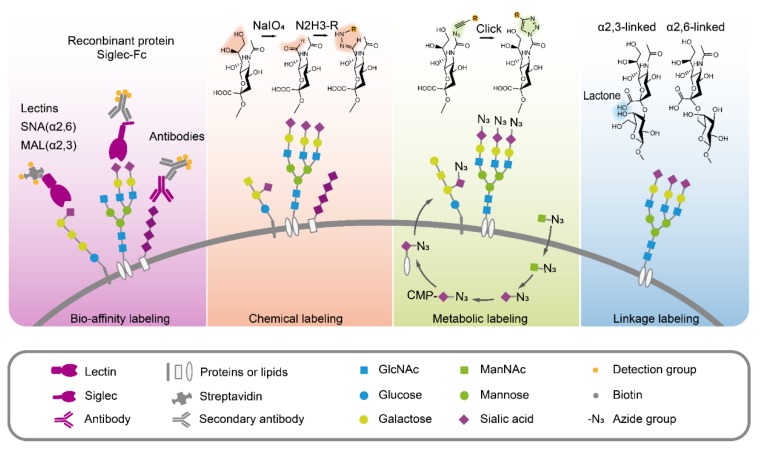
Different strategies for sialic acid analysis.

Since sialic acids have important functions in pathology and physiology, accurate, simple and rapid methods for sialic acid quantification and analysis have been developed, as summarized in Figure 3. 

### 3.1. Sialic Acids Quantification

The high sensitivity of sialic acids in serum or plasma as a tumor marker has been reported in various cancerous conditions [26]. Quantification of total sialic acids or glycolipid-bound sialic acids in serum is helpful to improve the accuracy of clinical diagnoses and therapies. Detection methods have been established for quantification of sialic acids, including colorimetric assay [99], fluorometric assay [100,101], high performance liquid chromatography (HPLC) [102,103], fluorescence [104,105,106], and mass spectrometry (MS) [107,108,109]. Among those methods, HPLC and MS are widely used [110,111]. Free sialic acids are easy to detect using the above methods but the polysialic acid and the sialic acids bound on glycoconjugates need to be released by acid hydrolysis or neuraminidase. For HPLC analysis, the free sialic acids react with reagents to form chromogen, which can be measured based on their spectral absorption. Sample preparation involves non-derivatization or derivatization. Derivatizations could stabilize sialic acid and significantly enhance the detection sensitivity. The fluorogenic reagent, 1,2-diamino-4,5-methylenedioxybenzene (DMB) is commonly used to derivatize sialic acids. The DMB derivatives of sialic acids can be quantitatively analyzed by an HPLC system equipped with a fluorescence detector or by liquid chromatography electrospray ionization-mass spectrometry. Chromatographic separation, in particular, allows the successful separation of the sialic acids from interfering compounds using separation columns such as ion-exchange. For LC-MS analysis, MS is usually used as the detector, following LC separation, and the sialic acid structures can be easily distinguished by the precursor and fragmentation mass on MS. In summary, sialic acid in a complex sample could easily be quantified by the HPLC and MS method, which could be applied in tumor research and diagnosis [26]. For example, sialic acid, quantified with fluorescence detection-HPLC in the IgM-enriched fraction, was significantly higher in cancer patients [112]. The sialylated proteins, as potentially cancer-associated proteins in serum, were quantified in prostate cancer using LC-MS/MS [113,114]. 

### 3.2. Sialic Acids Detection Through Bio-Affinity

Over the past decade, several bio-affinity-based approaches for directly detecting sialic acids and sialylglycans have been developed, including lectins, antibodies, and recombinant sialic acid-binding proteins [115]. Lectins are sugar-binding proteins that can specifically recognize glycans on glycoconjugates. *Sambucus nigra* lectin (SNA) and *Maackia amurensis* lectin (MAL) are commonly used to recognize the α2,6-linked and α2,3-linked sialic acid residues, respectively [116,117,118], in lectin blot [119], lectin microarray [120,121,122], histochemistry [123], fluorescent image and flow cytometry [56,124]. Using lectin MAL-1, α2,3-linked sialic acids were found to promote gastric cancer cell metastasis [125]. Detecting sialic acids using antibodies is a routine strategy. However, antibodies against sialic acid are normally generated from carbohydrate antigens, and are usually immunoglobulin M (IgM) with low binding affinity. Antibodies only exhibited better affinity for polysialic acid with long-chain polymer [110]. Thus, antibodies are not widely used for detecting sialic acids [115]. Alternatively, the sialic acid receptors fused with tags or fluorescent protein have also been used in sialic acid detection. For example, Siglecs fused with the Fc portion of human IgG1 (Siglecs-Fc) were applied to the detection of sialylglycans [126]. Those methods provided a simple way to directly detect and visualize sialic acids in cells or tissues and to better understand the relationship between sialic acids and tumor progression. 

### 3.3. Chemical Modification to Label Sialic Acids

Since the side chain at C7, C8, C9 of sialic acid is specifically sensitive to periodate oxidation under mild conditions, periodate oxidation has been designed to generate aldehyde groups from the sialic acids. This reaction selectively oxidizes the glycerol side chain of sialic acid to form a 7-aldehyde sialic acid derivative, which further can be linked to hydrazine beads or fluorescence probes and used for detecting the sialic acids on living cell surface [116,127] and for glycoproteomic research. In this method, periodate oxidation imine ligation was improved by the presence of aniline for better efficiency [128]. Moreover, the aldehyde group also offers a possible way to quantify the sialoglycoconjugates. A rapid periodate oxidation of the sialic acid side chain in common core structures of gangliosides was carried out using NaIO_4_ treatment followed by ligation with a carbonyl-reactive isobaric tandem mass tag (TMT) and subsequent LC-MS/MS analysis. The TMT tag improved the ionization efficiency of complex gangliosides and could simultaneously quantify up to six samples, as well as the identification of glycan and lipid compositions in a single injection [129]. For *N*-glycans, triplex *md*SUGAR tags were developed for quantitative glycomics. *md*SUGAR tags could be easily linked to the reducing end of glycans and the additional aldehyde group introduced by mild periodate oxidation, which extend the mass difference and lower the requirement for resolving power [130]. In brief, a mild periodate oxidation reaction offers an efficient way to specifically and selectively oxidize the polyhydroxy chain of sialic acids to form the aldehyde group, which can be further linked to tags and fluorescence probes for enrichment or detection. Despite the potential side effects, the advantages of direct chemical labeling are still attractive. Samples from different sources such as cells, tissues, or biological fluids can be directly labeled after harvest at biocompatible reaction conditions with high ligation efficiency.

### 3.4. Sialic Acids Metabolic Glycan Labeling

As shown in Figure 1, the biosynthesis of sialic acids and sialyl glycoconjugates starts from UDP-*N*-acetylglucosamine (UDP-GlcNAc) enzymatic conversion. The exogenous sialic acid precursors, including ManNAc, sialic acid, and CMP-sialic acid, can be taken up and metabolized by mammalian cells, resulting in the high expression of sialic acids in cell surface sialoglycoconjugates. Due to the broader substrate tolerance of enzymes involved in sialylglycans biosynthesis, it is possible to use the sugar analogues with functional groups (e.g., ketones, azides, alkynes) for sialic acid metabolic labeling [131,132]. Theoretically, *N*-azido-acetylmannosamine (ManNAz) can be used in living cell surface glycans labeling. However, the absence of an efficient transporter and hydrophilic property limit ManNAz entering cells. Instead, the hydrophobic per-*O*-acetylated ManNAz (Ac_4_ManNAz) was used for sialic acid metabolic labeling [133]. Ac_4_ManNAz can easily cross the cell membrane. After Ac_4_ManNAz have entered the cell, the extra acetyl groups are deacetylated by cytosolic esterases, and glycan biosynthetic enzymes can utilize these deprotected sugar derivatives [134]. Therefore, Ac_4_ManNAz has been widely used in the cell surface sialic acid labeling, such as the sialoglycoprotein expression upon megakaryocytic differentiation [135], imaging the glycosylation state of cell surface glycoproteins [136], single cell [137] or selective cell [138] metabolic glycan labeling, imaging sialyl glycomics during zebrafish development [139]. Amazingly, using an Ac_4_ManNAz metabolic labeling strategy, sialylated *N*-glycans on a select group of small noncoding RNAs (glycoRNAs), including Y RNAs, have been observed in mammalian cells [140], which may update our knowledge towards biochemistry, as we already know [141]. It is worth noting that per-*O*-acetylated monosaccharides can spontaneously react with numerous cysteines in proteomes with non-enzymatic catalysis, resulting in abnormal S-glycosylation [142]. In consideration of this condition, it is necessary to verify the results by specific glycosylation sites to exclude the S- glycosylation on cysteines when performing sialoglycoprotein research using Ac_4_ManNAz. Despite the defect, metabolic labeling has been widely applied to cell engineering, chemical tumor targeting, and tumor therapy [143,144,145]. In summary, these metabolic glycan labeling methods can be efficiently used for the tracking and visualization of sialic acids, glycoproteome profiling and identifications, and the studies of sialic acid-dependent ligand-receptor interactions.

### 3.5. Distinguishing the Sialic Acid Linkages

Sialic acids are frequently the terminal residues of glycan chains and are typically connected either by a α2,3- or α2,6-glycosidic bond to galactose (Gal) and/or *N*-acetylgalactosamine (GalNAc). Usually, lectins are used to recognize sialic acids with specific linkages. However, non-specific binding of lectin SNA and MAL makes the results unreliable. Considering the different spatial conformations, α2,3-linked sialic acid can form a stable lactone to the neighbor galactose, whereas α2,6-linked sialic acid cannot. This character makes it possible to differentiate the specific linkage of sialic acid on glycans through molecular weight. When sialylglycans were derivatized by dimethylamine, α2,3-linked sialic acid formed a stable amidation structure, which showed a mass shift of −0.984 Da, whereas α2,6-linked sialic acid formed a stable dimethyl amide and remained stable in ammonium hydroxide, and the dimethylamidation of α2,6-linked sialic changed glycans mass by +27.047 Da. The different mass shifts of α2,3- and α2,6-linked sialic acid derivatives can be distinguished by MALDI-TOF MS [146]. Using this strategy, the profiling of sialic acids on IgG glycopeptides [147,148], changes of sialic acid linkages in hypoxia condition of lung cancer A549 cells and in clinical ovarian cancer tissue [149,150], and the visualization of sialic acid on leiomyosarcoma tissues [151] have been successfully performed. In summary, this method offers an accurate way to explore the potential relationship between tumors and specific sialic acid linkages. 

## 4. Conclusions and Future Prospects

In this review, we summarized the possible functions of sialic acids in tumor development and progression. Sialic acids commonly locate at the terminal of glycans on the cell surface. The negative charge and hydrophilic property give the sialic acid coated cells unique characters. Hypersialylation is usually found in tumor tissues and is supposed to be a potential hallmark of cancer. Sialic acids enhance tumor progression at multiple levels by facilitating escape from immunological surveillance, angiogenesis, the formation of metastasis, and resistance to apoptosis and therapy. For this reason, the therapeutic approaches to modulate sialic acids and their receptors can be of high potency in tumor therapy [152,153,154]. As the coated sialic acids on tumor cells masked the antigenic sites of glycoproteins, removing sialic acids by sialidases is expected to become potential treatment. Recently, co-expression of the surface sialidase in chimeric antigen receptor T-cells (CAR-T cells) has proven to be effective against solid tumors [155]. The sialylation on IgG was reported to induce anti-inflammation activity and to enhance stability and effector functions in antibody therapy [153,154,156]. Innovatively, engineering the glycan modification of extracellular vesicles by removing sialic acids and inserting Lewis^Y^ glycans enhanced the antigen presentation to dendritic cells, which might act as a “vaccine” for tumor treatment [157]. The polysialic acid modified liposomes were used as a targeted drug delivery system to enhance anti-cancer efficiency [158]. Moreover, antibody against siglec-15, a sialic acid binding protein and T cell depressor, is expected to apply to cancer immunotherapy, offering another therapeutic tool for cancer patients resistant to current anti-PD-1/PD-L1 therapy [49]. 

Fortunately, owing to novel technologies and methods, increasing discoveries of sialic acid functions in physiology and pathology are being reported. In clinical studies, sialylation in human serum or saliva can be accurately detected using quantification methods. Notably, specific proteins, such as IgA1 with sialylation, were proved to relate to certain tumor occurrences [159]. Enhanced levels of sialic acids in clinical serum provided a promising biomarker and a reliable predictor for prostate cancer and its bone metastases [160]. In fundamental research, more labeling methods are innovated, such as the chemical labeling after mild periodate oxidation and the sialic acid precursors analogues metabolic labeling. In recent years, chemists have designed various sugar analogues containing functional groups, making it more easy and convenient to label and track sialic acids [132]. Among those sugar analogues, Ac_4_ManNAz is mostly used as the azido group can be easily linked to probes or beads through click chemistry reaction. Sialic acid metabolic labeling combined with high resolution LC-MS offers simple detection in sialic acid modification sites of a certain protein [161]. As a result, analyzing the sialic acid modification in specific proteins enables researchers to explain the relationship between sialic acid modification and tumor progression in depth. We believe, in the near future, treatment such as reducing sialic acids or targeting the inhibitory sialic acid receptors, together with the immunotherapy, will release and rescue more patients suffering from cancer. 

## Abbreviations

Ac_4_ManNAz	Per-*O*-acetylated *N*-azido-acetylmannosamine;
AGF	Angiogenic growth factor;
CMAS	Cytosine 5′-monophosphate *N*-acetylneuraminic acid synthetase;
CAR-T cell	Chimeric antigen receptor T-Cell;
CMAH	Cytidine monophosphate *N*-acetylneuraminic acid hydroxylase;
CMP-Neu5Ac	Cytidine-5′-monophospho-*N*-acetylneuraminic acid;
EGF	Epidermal growth factor;
EGFR	Epidermal growth factor receptor;
EMT	Epithelial-mesenchymal transition;
ERK	Extracellular signal–regulated kinases;
FAK	Focal adhesion kinase;
Fc	The fragment crystallizable region of antibody;
GalNAc	*N*-acetylgalactosamine;
GNE	Hydrolyzing UDP-GlcNAc 2-epimerase/ManNAc-6-kinase;
HER	Human epidermal growth factor receptor;
HPLC	High performance liquid chromatography;
IgA/G/M	Immunoglobulin A/G/M;
ITIM	Immunoreceptor tyrosine based inhibitory motif;
LC-MS	Liquid chromatography–mass spectrometry;
LSA	Glycolipid-bound sialic acids;
MAL	*Maackia amurensis* lectin;
MALDI-TOF MS	Matrix-assisted laser desorption/ionization time of flight mass spectrometry;
ManNAc	*N*-acetyl-D-mannosamine;
MAP	Mitogen-activated protein;
MS	Mass spectrometry;
NAL	*N*-acetylneuraminate lyase;
NANP	Neu5Ac-9-phosphate phosphatase;
NANS	Neu5Ac 9-phosphate synthase;
NCAM	Neural cell adhesion molecule;
NEU	Neuraminidase; sialidase; acetylneuraminyl hydrolase;
Neu5Ac	*N*-acetylneuraminic acid;
Neu5Gc	*N*-glycolylneuraminic acid;
NK cell	Natural killer cell;
PD-1/PD-L1	Programmed cell death protein 1/ligand 1;
PECAM	Platelet endothelial cells adhesion molecule;
PEP	Phosphoenolpyruvate;
Siglec	Sialic acid-binding immunoglobulin-like lectins;
SNA	*Sambucus nigra* lectin;
TMT	Tandem mass tag;
TSA	Total sialic acid;
UDP-GlcNAc	Uridine diphosphate *N*-acetylglucosamine;
VEGF	Vascular endothelial growth factor;
VEGFR	Vascular endothelial growth factor receptor.
